# The Role of Parental Verbal Threat Information in Children’s Fear Acquisition: A Systematic Review and Meta-analysis

**DOI:** 10.1007/s10567-024-00485-4

**Published:** 2024-05-24

**Authors:** Cosima Anna Nimphy, Vasiliki Mitrou, Bernet M. Elzinga, Willem Van der Does, Evin Aktar

**Affiliations:** 1 Department of Clinical Psychology, Leiden University, Leiden, The Netherlands; 2 Leiden Institute for Brain and Cognition (LIBC), Leiden, The Netherlands; 3 Leiden University Treatment Center (LUBEC), Leiden, The Netherlands

**Keywords:** Verbal threat, Fear, Instructional learning, Children, Parental anxiety, Child anxiety

## Abstract

Children can acquire fears of novel stimuli as a result of listening to parental verbal threat information about these stimuli (i.e., instructional learning). While empirical studies have shown that learning via parental information occurs, the effect size of parental verbal threat information on child fear of a novel stimulus has not yet been measured in a meta-analysis. We conducted a systematic review and meta analysis to assess the effect of parents’ verbal statements on their children’s fear acquisition. Additionally, we explored potential moderators of this effect, namely, parent and child anxiety levels, as well as child age. WebOfScience, Pubmed, Medline, and PsycINFO were used to identify eligible studies that assessed children’s (30 months to 18 years old) fear of novel stimuli after being exposed to parental verbal threat information. We selected 17 studies for the meta-analysis and 18 for the systematic review. The meta-analysis revealed a significant causal effect of parental verbal threat information on children’s fear reaction towards novel stimuli [*g* = 1.26]. No evidence was found for a moderation of verbal learning effects, neither by child or parent anxiety levels nor by child age. The effect of parents’ verbal threat information on children’s fear of novel stimuli is large and not dependent on anxiety levels or child age.

**Supplementary Information:**

The online version contains supplementary material available at 10.1007/s10567-024-00485-4.

## Introduction

Anxiety disorders are among the most prevalent clusters of mental disorders in children and adolescents (Bandelow & Michaelis, 2015; Kessler et al., 2005; Remes et al., 2016). Individuals with anxiety disorders suffer from excessive worry and anxiety, which impairs their daily functioning, including their social life or academic performance (Quilty et al., 2003). The disorder often takes a chronic course, meaning that, without successful intervention, it tends to prevail (Beesdo et al., 2009; Keller et al., 1992). In order to develop successful interventions, it is important to gain insight into the mechanisms that play a significant role in how anxiety disorders develop.

Anxiety runs in families (Beidel & Turner, 1997; Eley et al., 2015; Hudson et al., 2011). Children with parents who had or have an anxiety disorder have a two- to threefold risk for developing an anxiety disorder, compared to children of parents without anxiety (Lawrence et al., 2019; Telman et al., 2018). To reduce this increased risk of anxiety in the offspring it is crucial to understand how the anxiety transmission unfolds in the family. Many studies have assessed the impact of both genetic and/or environmental influences in the familial aggregation of anxiety disorders (Eley et al., 2015; Gregory & Eley, 2007; Hettema et al., 2001). Genetic transmission explains approximately one-third of the variance in child anxiety (Hettema et al., 2001). This leaves the majority of variance unexplained and attributed to environmental factors, alone and in interaction with genetic factors (Gregory & Eley, 2007). This is in line with a children-of-twins design study, where the relative influence of genetic and environmental factors was investigated and showed that environmental factors predominantly accounted for the parent–child transmission of anxiety (Eley et al., 2015). This calls for research that elucidates the mechanisms involved in this transmission.

Children can acquire fears via others, including parents (also known as social fear learning, Rachman, 1977; Olsson et al., 2007) in two ways. Firstly, children can acquire fear of a novel stimulus via modeling: observing others being fearful towards that novel stimulus (also known as vicarious fear learning). Within the family context, children can for example learn to fear a novel animal as a result of being exposed to parents’ anxious responses to that animal (Murray et al., 2008). This vicarious fear transmission starts as early as in infancy, as children start seeking out information about novel stimuli from parents between 10 and 14 months of age (so called social referencing, Feinman, 1982; Nimphy et al., 2023b). Secondly, children can learn to be fearful of a novel stimulus when they receive verbal information from others about the threatening/anxiety-provoking properties of this stimulus (also known as verbal (threat) information learning or instructional learning, Olsson et al., 2007; Muris & Field, 2010). During early childhood, when children learn to speak, verbal information about novel stimuli from parents becomes especially salient (Berman, 2004). Rachman suggests that verbal information from parents and peers during childhood is the origin of most fears in daily life (Rachman, 1977). Findings of cross-sectional and longitudinal studies that assessed origins and potential mechanisms underlying childhood anxiety suggest a role of parental verbal threat information (Fliek et al., 2017, 2019; Ollendick & King, 1991). Moreover, in a review, Muris and Field (2010) argued that there is “clear support for the notion that the verbal provision of threat information may have fear-enhancing effects in children”. Therefore, in this meta-analysis, we focus on this verbal information-learning pathway by summarizing the empirical evidence on child acquisition of fear and anxiety via parental verbal threat information.

Besides the line of research investigating whether parental verbal threat information is related to child anxiety (symptoms), biased cognition, or general fearfulness, two distinct lines of research have studied child fear acquisition of *specific* novel stimuli via parental verbal threat information. The first line of studies focuses on typically developing children and employs experimental designs, where parents are instructed/trained to express specific verbal information towards novel stimuli (i.e., Aktar et al., 2022; Bell et al. 2015; Remmerswaal et al., 2013). The second line of studies relies on naturalistic observations of anxious and non-anxious parents with their children, that investigate the relationship between parental verbal threat information about a novel stimulus and child fear responses to the stimulus in daily life (i.e., Nimphy et al., 2023a; Radanović et al., 2021; Remmerswaal & Muris, 2011; Setiawan et al., 2018; Uy et al., 2022). While the first line may enable us to draw causal inferences, the second line aims to capture anxiety transmission in daily life. Our meta-analysis will examine both complementary lines.

Importantly, parent-to-child transmission of fear serves an evolutionary adaptive purpose, namely helping children in recognizing and avoiding dangerous situations, to enhance their chances of survival (Feinman, 1985). However, parents with an anxiety disorder, who experience excessive fear and have a tendency to overestimate threat (American Psychiatric Association, 2013), may inadvertently express anxiety—even in the absence of a threat. Parents with higher trait anxiety make more negative statements about a novel stimulus to their children than parents with lower levels of trait anxiety (Muris et al., 2010). Over time, children of anxious parents may develop heightened attention to threat signals or interpret the signals in a more negative manner (Aktar, 2022; Creswell et al., 2010). Consequently, the influence of fear expressions on a child’s acquisition of novel stimuli might be more pronounced in children of anxious parents than in those with non-anxious parents.

Besides the role of parental anxiety, previous studies also investigated child characteristics such as temperament, general fearfulness, or anxiety symptoms as a potential moderator in the parent-to-child transmission of fear, possibly strengthening the effect that parent verbal anxiety expressions have on their children’s fear acquisition (Muris & Field, 2010; Percy et al., 2016). For example, child behavioral inhibition (BI) is an important risk factor for developing social anxiety (see Clauss & Blackford, 2012). Moreover, BI was proposed to be a marker of enhanced vulnerability to environmental stressors, including parental anxiety expressions (Belsky & Pluess, 2009; Ingram & Luxton, 2005; Nigg, 2006). Nevertheless, findings regarding the moderating role of child anxiety dispositions in parent-to-child fear transmission are mixed, allowing no firm conclusion about a potential moderating role (Muris & Field, 2010).

The impact of the parental verbal threat information on child fear acquisition of novel stimuli might also depend on the child’s developmental stage, with children being more affected by parental anxiety expressions in earlier stages. As children develop increasingly advanced cognitive and emotional abilities, they gradually become more emotionally independent from their parents as they age (Morris et al., 2007). In line with this idea, one study that investigated the relationship between parental verbal threat information on children’s fear of Covid-19, suggests that younger children might be more sensitive to parental verbal threat information (Uy et al., 2022). They argue that older children may have greater emotion regulation capacity, which might dampen the impact of parental verbal threat information, compared to younger children. Younger children might also depend more heavily on their parents as sources of information than adolescents. However, this empirical finding still has to be replicated.

Currently, knowledge on the parent–child transmission of fear through parental verbal threat information and the moderating roles of child temperament and parental anxiety is based on narrative and systematic reviews (Emerson et al., 2019; Muris & Field, 2010; Percy et al., 2016). These reviews have concluded that parent–child transmission of fear via verbal threat information is a significant factor contributing to child acquisition of fear and anxiety. More specifically, the reviews argue that fear acquisition as a result of verbal threat information can manifest in children’s fearful and anxious cognitions (Muris & Field, 2010; Emerson et al., 2019), heart rate (Muris & Field, 2010) and avoidant behavior to novel stimuli (Muris & Field, 2010; Percy et al., 2016). Taken together, the findings summarized in these reviews also suggest the effect of verbal threat information on children’s cognitions, implicit associations, and behavior is noticeable for up to 6 months (Muris & Field, 2010).

This *meta-analysis* aims to combine the available evidence from empirical studies to calculate the effect size of the relationship between verbal threat information and child fear and avoidance of a novel stimulus. In line with previous studies (Muris & Field, 2010), we included studies that assessed child fear or anxiety with behavioral (i.e. avoidance), physiological (i.e. elevated heart rate), or cognitive (i.e. fear belief) measures. We expected that verbal threat information from parents is positively correlated with childrens’ fear or avoidance towards a novel stimulus. Furthermore, we explored whether the relationship between parental verbal threat information and child fear of novel stimuli is stronger for children of parents with higher anxiety levels/an anxiety disorder, children with higher levels of anxiety dispositions, and younger children. By gaining more specific insights into the verbal threat information pathway, we aim to improve our theoretical understanding on fear learning mechanisms in childhood and possible practical applications in prevention efforts.

## Methods

### Protocol and Registration

We followed the PRISMA guidelines, proposed by Moher and colleagues (2009) (see supplementary material for the PRISMA Checklist). Furthermore, this study was preregistered on the Open Science Framework (OSF) (https://doi.org/10.17605/OSF.IO/7THK5).

### Search Strategy

WebOfScience, PsycINFO, Embase (Medline) and PUBMED databases were searched to identify relevant studies. The database search included studies up to the 10th of November 2023 (date of search). The final search term was: (child* OR adolescent* OR toddler* OR teenager*) AND (parent* OR mother* OR father* OR caregiver* OR guardian*) AND ((transmission OR acquisition* OR (“observation* learning”) OR (“verbal threat*”) OR conditioning) AND (fear* OR avoid* OR anxi*)) AND (verbal OR instruction OR information). For an overview on the construction of the search term, see Supplementary Material A. Twenty percent of the screening process for inclusion was double-coded by an independent reviewer to establish interrater reliability of identifying relevant studies. The interrater agreement on the inclusion of studies was high, with Cohen’s kappa of 0.85. Inconsistencies were resolved through consensus. After the identification of relevant articles, all duplicates were removed. Next, in a secondary screening step, additional articles identified through the reference lists were added (*n* = 49). These articles were then also screened.

### Inclusion and Exclusion Criteria

This meta-analysis included published studies that measured fearful or anxious responses in human children (between 30 months and 17 years) after exposure to parental verbal responses of fear or anxiety. These studies had to assess child fear or anxiety with behavioral (i.e., avoidance), physiological (i.e., elevated heart rate), or cognitive (i.e., fear belief) measures. We included studies, which investigated how parent’s verbal fear or anxiety information/instruction towards a stranger, novel object, or situation can shape their children’s reaction to the same ambiguous stranger, object, or situation. Studies that investigate only the non-verbal transmission of anxiety or fear were excluded (i.e., vicarious learning, also known as modeling). We excluded studies, which only investigated children who are hearing impaired, or have neurodevelopmental delays, as it could interfere with verbal fear transmission. The meta-analysis only included studies published in English. To be included in the meta-analysis, the extracted statistical information in a study’s result section should allow for calculation of effect sizes for at least one outcome measure.

### Data Extraction

Two reviewers independently extracted relevant information from identified studies. Inconsistencies were resolved through consensus. The data that was extracted are demographic information (i.e., age of the participating parents and children, occupation/socio-economic status (SES), ethnicity, gender, and study location) and methodological characteristics (i.e., study design, number of outcome variables, measurement tools for predictor and outcome variable number of outcome variables, and reliability estimates). Additionally, we extracted means, standard deviations, correlation coefficients, effect sizes, and corresponding 95% Confidence Intervals (CI) of the variables and associations of interest. Variables of interest are child anxious/fearful expressions, parent anxious/fearful verbal (and nonverbal) expressions, parent psychopathology, child temperament or anxiety disposition, and type of stimulus (i.e., social versus non-social). All effect sizes were converted to Hedges’ *g*, as most studies provided relevant statistical information about the experimental and control condition. For studies that reported insignificant findings without providing relevant statistical information beyond the sample size and non-significance, we assumed a *p*-value of 0.5 (one-directional) to calculate the effect size. This results in an effect size of 0 with the accompanying variance (see Dusseldorp et al., 1999). This method was used as excluding the insignificant finding from analyses would inflate the effect sizes. We only assessed the effect sizes for the moderators if a subset consisted of at least four studies (*k* ≥ 4) (Bakermans-Kranenburg et al., 2003).

### Statistical Analyses

We carried our analyses with the metafor package in R. Statistical significance of the pooled Hedges’ *g* was assessed using a *Z*-test at *p* < 0.05. Heterogeneity between the studies was theoretically anticipated and thus we chose the random effects model. However, we still checked for heterogeneity using the *Q*-test. A two-tailed *p* significance test was used with statistical significance, if *p* < 0.05. We corrected the effect sizes to a weighted effect size (corrected for unequal *n*’s) and checked for publication bias with a funnel plot. In case of publication bias, a trim and fill method was applied. To detect effect size outliers, we checked whether the standardized residual *z* > 3.

#### Quality and Bias Assessment

The methodological quality of the included articles was checked using a checklist (results presented in Table S1) based on the Cochrane Collaboration tool (ROB2) and adapted to our study design (for details on Quality assessment, see Nimphy et al., 2023b). Examples of these assessment criteria are the reliability of the predictors and outcome measures, as well as how transparent the results are reported.

## Results

**Fig. 1:**
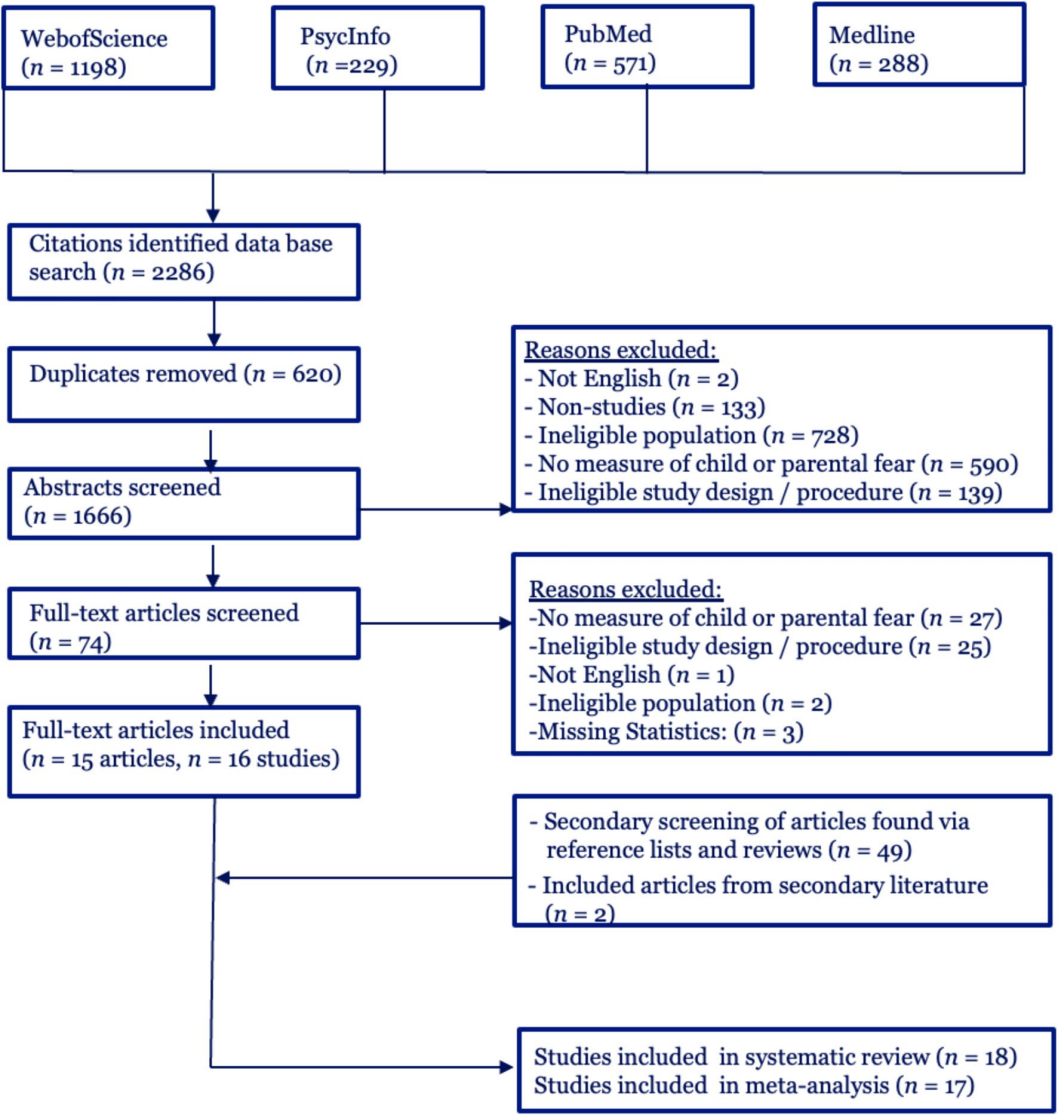
Flow diagram

Our search term yielded overall 2286 hits across WebofScience, PsycInfo, Pubmed, and Medline. After the removal of 620 duplicates, we screened 1666 studies and included 15 articles. During the secondary screening process, we screened the abstracts of 49 and the full text of 25 studies and included two more studies. The screening process and reasons for exclusions at each stage are presented in the flow diagram (Fig. 1).

### Overview of Studies

The study characteristics of the studies included in the systematic review and meta-analysis can be found in Table 1. We included 18 studies (from 17 articles) in the systematic review and 17 studies (from 16 articles) in the meta-analysis. The study of Reider et al. (2022) included two experiments with two independent samples. Therefore, we added them as separate samples in our analyses. Furthermore, two studies that were included in the systematic review (Aktar et al., 2014, 2018) contained analyses of the same children at different developmental stages. For the meta-analysis, we chose to include only data from the first study (Aktar et al., 2014), as it contained the data from a larger sample size. The quality ratings of all studies included in the systematic review and meta-analysis ranged from 71.4% to 100%, with a mean percentage of 92.97% (for the quality rating per study see Table S1 in supplementary material B).

**Table 1:** Overview of studies included in systematic review

Study	General characteristics			Child characteristics				Parent characteristics							
	Journal	Location	Design	*N* (*n* after exclusion)	% Girls	Range in years (mean age)	Child anxiety disposition/BI	*N* (*n* after exclusion)		% Mothers	Mean age (years)	SES	Ethnicity % CAU	Anxiety	
Aktar et al. (2014)	Journal of Child Psychology and Psychiatry	Europe, The Netherlands	Correlational	117 (subsample of Aktar et al., 2013)	55	(2.5)	Assessed with *LAB-TAB*	234 (232)		50.43	34	Moderate to high	92.31 (Parent)	Assessed with *ADIS*: 55.56%	
Aktar et al. (2018)	Journal of Clinical Child and Adolescent Psychology	Europe, The Netherlands	Correlational	111 (106) (subsample of Aktar et al., 2013)	52	(4.5) data of 2.5-year-olds’ not included	Assessed with *LAB-TAB*	222 (212) 212		50	37.58	Moderate to high	93.23 (Parent)	Assessed with *ADIS*: 7.66% (current), 9.01% (lifetime)	
Aktar et al. (2022)	Developmental Psychobiology	Europe, The Netherlands	Experimental	68	50	4.03–6.65 (5.27)	Assessed with *BIQ*, *CBQ* and *SCARED*	136 online survey and 68 for lab visit		50	37.5	Moderate to high	86.75 (Parent) 91% (Child)	Assessed with *SCARED-A* (subscale social anxiety)	
Becker and Ginsburg (2011)	Child Psychiatry and Human Development	North America, USA	Correlational	75	52	6–14 (9.03)	NA	75		100	39.91	High	78.7 (Child)	Assessed with *ADIS*: 50.67% (current anxiety)	
Bell et al. (2015)	Australian Journal of Psychology	Oceania, Australia	Experimental	122	54	7–12	Diagnosis assessed with: ADIS-C/P-IV, Severity with: *SCAS-C*: 44.23 (anxious) 26.67 (non-anxious)	121		100	NA	NA	NA	Assessed with *STAI-T*: 55.37% high trait anxiety, Overall *M* = 35.23	
Bosmans et al. (2015)	Parenting	Europe, Belgium	Experimental	60	67	9–12 (10.47)	Assessed with *STAI-C*: 32.32	60		100	NA	NA	“All mothers were European”	NA	
Burstein and Ginsburg (2010)	Behavior Research and Therapy	North America, USA	Experimental	25	44	8–12 (9.24)	Assessed with *SCARED*: 10.12 *CBCL*: 46.32 (internalizing)	25 (24)		52	41.8	Moderate to high	76 (Child)	Assessed with *ASR*: *M* = 42.88 (internalizing)	
Muris et al. (2010)	Behavior Research and Therapy	Europe, The Netherlands	Experimental	88	48	8–13 (10.28)	Assessed with *FSSC-R*: 39.49	88	81.82		40.4	NA	84.1 (Parent)	Assessed with *STAI*: *M* = 34.92	
Muris et al. (2013)	Behavior Therapy	Europe, The Netherlands/Germany	Experimental	60	50	8–12 (10.65)	Assessed with *FSSC-R*: 40.85	60	100		NA	NA	88.33 (Child)	NA	
Nimphy et al., (2023a)	Journal of Adolescence	Europe, The Netherlands	Correlational	195	58	8–18 (14.23)	Assessed with *BIS*: 2.26	193	76		47.82	Moderate to high education	100 “Dutch parent–child dyads”	Assessed with *SCARED-A*: *M* = 0.32	
Radanović et al. (2021)	Frontiers in Psychology	Europe, Serbia	Correlational	376	60	7–19 (12.77)	Assessed with *FSSC-R*: 2.46	376	NA		42.9	NA	NA	Assessed with *STICSA*: *M* = 1.70 (cognitive) *M* = 1.40 (somatic)	
Reider et al. (2022) Study 1	Developmental Psychology	North America, USA	Experimental	27	44.4	4.05–6.83 (5.33)	NA	27	81.48		NA	Moderate to high	74.1 (Parent)	NA	
Reider et al. (2022) Study 2	Developmental Psychology	North America, USA	Experimental	54	50	3.67–7.07 (5.52)	NA	54	81.48		NA	Moderate to high	61.1 (Parent)	NA	
Remmerswaal et al. (2010)	Journal of Anxiety Disorders	Europe, The Netherlands	Experimental	52	52	9–12 (10.6)	Assessed with *FSSC-R*: 40.83	52 (50)	100		42.9	NA	95.74 (Parent and children)	Assessed with *STAI*: *M* = 33.60	
Remmerswaal and Muris (2011)	Journal of Anxiety Disorders	Europe, The Netherlands	Correlational	223 (220)	53	7–12 (9.97)	Assessed with *FSSC-R*: 7.63	347 (342)	58.21		43.1	NA	NA	Assessed with *FSSC-R* (medical fear): *M* = 6.70	
Remmerswaal et al. (2013)	Behavior Therapy	Europe, The Netherlands	Experimental	47	66	8–12 (10.55)	Assessed with *FSSC-R*: 31.95	47	100		41.8	NA	70.2 Dutch (Parents and Children)	Assessed with *STAI-Y2*: *M* = 47.77	
Setiawan et al. (2018)	European Journal of Dentistry	Southeast Asia, Indonesia	Correlational	550	NA	3–6	NA	NA	NA		NA	NA	NA	NA	
Uy et al. (2022)	Developmental Psychobiology	North America, USA	Correlational	283	44.2	5.5–17 (10.17)	NA	283	97.9		NA	Moderate to high	66.7	NA	

For journal: name of journal in which the article was published. For location: study location. For child characteristics: *N* number of children in the sample, *BI* behavioral inhibition/temperament, *CAU* Caucasian. For parent characteristics: *N* number of parents in the sample, *SES* socio-economic status, *CAU* Caucasian; *Anxiety* percentage of parents who have anxiety based on diagnostic tools or questionnaires or mean anxiety symptoms

#### Systematic Review

The study and sample characteristics are presented in Table 1. The studies differed in (1) design, (2) child fear index, (3) parental message type, and (4) stimulus type. Below we address each of these in detail.

First, concerning the study design, from the 18 studies included in this systematic review, 8 had a correlational design, whereas 10 had an experimental design. In the correlational designs, parental verbal threat information regarding novel stimuli were not manipulated/trained by the experimenter, but observed during a social referencing paradigm with parents and their children or assessed in their daily life. Of the 18 studies, 12 included a measure of parental anxiety symptoms or diagnosis, 3 studies included clinical parent samples, consisting of 16.67–55.56% of parents with an anxiety disorder (Aktar et al., 2014, 2018; Becker & Ginsburg, 2011), whereas 9 studies assessed anxiety (symptoms) in community samples of parents, reporting no or low anxiety scores (Aktar et al., 2022; Bell et al., 2015; Burstein & Ginsburg, 2010; Muris et al., 2010; Nimphy et al., 2023a; Radanović et al., 2021; Remmerswaal & Muris, 2011; Remmerswaal et al., 2010, 2013). Finally, 13 studies of these 18 studies assessed child anxiety dispositions (Aktar et al., 2014, 2022; Bell et al., 2015; Bosmans et al., 2015; Burstein & Ginsburg, 2010; Muris et al., 2010, 2013; Nimphy et al., 2023a; Radanović et al., 2021; Remmerswaal & Muris, 2011; Remmerswaal et al., 2010, 2013; Setiawan et al., 2018).

Second, there were also differences across studies in how child fear was operationalized (overview can be found in Table 2). From the 18 included studies, 12 studies primarily assessed child fear with a cognitive measure, specifically self-reported fear beliefs towards the novel stimulus. In two studies child reactions were assessed with just a behavioral measure of fear and avoidance (i.e., facial, vocal, and verbal expressions of fear). Four studies investigated child fear with both cognitive and behavioral measures. Three studies reported mean interobserver reliability (ICC or Cohen’s kappa) for the behavioral coding of observed child fear and avoidance (Aktar et al., 2014, 2018, 2022), which ranged from 0.87 to 0.93, and were classified to be of high interrater reliability. Twelve studies reported reliability for child cognitive fear indices, ranging from 0.49 to 0.97.

**Table 2:** Overview of the reviewed studies’ approach to measuring parental-verbal communication

Study	Type of parental message manipulated/assessed	Type of stimulus	Specifically	Assessment method	Specifically
Aktar et al. (2014)	Non-verbal and verbal	Social and non-social	Stranger and toy	Behavioral Measure: Fear and avoidance	Fear: intensity and frequency of facial, bodily, and vocal/verbal expressions of fear on a scale from 1 to 5 Avoidance: child attempt to gaze away, turn away or hide from stimuli on a scale from 1 to 5 Fear and Avoidance were combined into one measure
Aktar et al. (2018)	Non-verbal and verbal	Social and non-social	Stranger and toy	Behavioral Measure: Fear and avoidance	Fear: intensity and frequency of facial, bodily, and vocal/verbal expressions of fear on a scale from 1 to 5 Avoidance: child attempt to gaze away, turn away or increase distance from/ignore stimuli on a scale from 1 to 5
Aktar et al. (2022)	Verbal only	Social	Stranger	Behavioral Measure: Fear and avoidance	Fear: frequency and duration of facial, bodily, and vocal/verbal expressions of fear on a scale from 1 to 5 Avoidance: child attempt to gaze away, turn away, or increase distance from stranger, by walking away or hiding behind the parent on a scale from 1 to 5. Fear and Avoidance were combined into one measure
				Cognitive Measure: Fear and Avoidance	Fear: Attention Bias (Visual Search Task) Fear/Avoidance: Self-report Fear Beliefs Questionnaire (FBQ) from 1 (no, not at all) to 5 (yes, definitely)
				Physiological Measure: Fear	Fear: heart rate, beats per minute
Becker and Ginsburg (2011)	Non-verbal and verbal	Social	Videotaped Speech	Cognitive Measure: Distress	Distress: self-evaluation of distress on a scale from 1 to 5
Bell et al. (2015)	Verbal only	Non-social	Animal	Behavioral Measure: Avoidance	Avoidance: Distance in cm between Lego figure (representing child or child’s family) and animal
				Cognitive Measure: Fear	Fear: Self-report on a scale from 0 (no fear) to 10 (extreme fear)
Bosmans et al. (2015)	Verbal only	Non-social	Animal	Behavioral Measure: Avoidance Cognitive Measure: Fear and Avoidance	Avoidance: Latency time putting the hand in box with animal (Touch Box Task) Fear/Avoidance: Self-report FBQ from 1 (no, not at all) to 5 (yes, definitely)
Burstein and Ginsburg (2010)	Non-verbal and verbal	Non-social	Spelling test	Cognitive Measure: Anxiety and Avoidance	Anxiety: Self-report C-FAT anxiety about the test from 0 (not at all) to 4 (extremely) Avoidance: Self-report C-FAT on desired avoidance from 0 (not at all) to 4 (very much)
Muris et al. (2010)	Verbal only	Non-social	Animal	Cognitive Measure: Fear and Avoidance	Fear/Avoidance: Self-report FBQ from 1 (no, not at all) to 5 (yes, definitely)
Muris et al. (2013)	Non-verbal and verbal	Non-social	Animal	Cognitive Measure: Fear	Fear: Self-report on a scale from 1 (no, not at all) to 5 (yes, absolutely)
Nimphy et al., (2023a)	Verbal only	Non-social	Virus	Cognitive Measure: Fear	Fear: Self-report Fear of COVID-19 (FCQ) on a scale from 1 (not true) to 4 (very true)
Radanović et al. (2021)	Verbal only (for analysis)	Non-social	Virus	Cognitive Measure: Fear	Fear: Self-report Fear of COVID-19 for Children (FC19Q-C) from 1 (strongly disagree) to 5 (strongly agree)
Reider et al. (2022) Study 1	Verbal only	Non-social	Animal	Cognitive Measure: Fear and Avoidance	Fear/Avoidance: Self-report FBQ from 1 (no, not at all) to 5 (yes, definitely)
Reider et al. (2022) Study 2	Verbal only	Non-social	Animal	Cognitive Measure: Fear and Avoidance	Fear/Avoidance: Self-report FBQ from 1 (no, not at all) to 5 (yes, definitely)
Remmerswaal et al. (2010)	Verbal only	Non-social	Animal	Cognitive Measure: Fear and Avoidance	Fear: Wason Selection Task Fear/Avoidance: Self-report FBQ from 1 (no, not at all) to 5 (yes, definitely)
Remmerswaal and Muris (2011)	Verbal only	Non-social	Virus	Cognitive Measure: Fear	Fear: Self-report Fear of the Swine Flu (FSFQ) from 1 (not true) to 4 (very true)
Remmerswaal et al. (2013)	Verbal only	Non-social	Animal	Behavioral Measure: Avoidance Cognitive Measure: Fear and Avoidance	Avoidance: Latency time putting the hand in box with animal (Touch Box Task) Fear/Avoidance: Self-report FBQ from 1 (no, not at all) to 5 (yes, definitely)
Setiawan et al. (2018)	Non-verbal and verbal	Non-social	Dental fear	Cognitive Measure: Fear and Avoidance	Fear/Avoidance: Children Fear Survey Schedule-Dental Subscale (CFSS-DS)
Uy et al. (2022)	Verbal only	Non-social	Virus	Cognitive Measure: Fear and Avoidance	Fear/Avoidance: Self-report Fear of Illness and Virus Evaluation (FIVE) from 0 (not afraid at all) to 3 (afraid all the time)

Third, the variation in the delivery form of parental verbal expressions of fear towards novel stimuli in these studies can be categorized into (1) verbal messages only (such as “this is scary, right?”) and (2) combined nonverbal and verbal messages (also including nonverbal expressions of anxiety such as fidgeting). Regarding the correlational studies, we can only categorize how parental expressions of fear/threat were assessed but not delivered, whereas we can categorize how parental fear expressions were delivered in the experimental studies into (1) only verbal or (2) combination of verbal and nonverbal expressions. Out of 18 studies, 13 studies fall in the first category, whereas 5 studies were in the second. Furthermore, in experimental designs, the threat condition was defined as fearful/anxious verbal messages, whereas the control condition could either consist of parental neutral verbal expressions or positive verbal expressions. Three studies reported mean interobserver reliability (ICC or Cohen’s kappa) for coded parent variables (Aktar et al., 2014, 2018; Becker & Ginsburg, 2011) which ranged from 0.68 to 0.88. One study reported 100% agreement for coded parent variables (Aktar et al., 2022).

Fourth, the stimuli that were paired with parental verbal information differed across studies and can be categorized into social and non-social stimuli. Social stimuli entailed exposure to a stranger, whereas non-social stimuli entailed exposure to animals, toys, and novel situations. The majority of studies (*k* = 13) included non-social stimuli, whereas three studies used only social stimuli in their social referencing paradigms (Aktar et al., 2022; Becker & Ginsburg, 2011; Burstein & Ginsburg, 2010), and two studies included both social and non-social stimuli (Aktar et al., 2014, 2018).

#### Meta-Analysis

Overall, of the 18 studies included in the systematic review, 16 studies entailing 17 samples were also included in the meta-analysis. Seven studies had a correlational design (Aktar et al., 2014; Becker & Ginsburg, 2011; Nimphy et al., 2023a; Radanović et al., 2021; Remmerswaal & Muris, 2011; Setiawan et al., 2018; Uy et al., 2022) and the remaining ten studies had an experimental design. Thirteen studies entailed non-social stimuli, three had only social stimuli, and one study included both social and nonsocial stimuli (Aktar et al., 2014). Not every study reported multiple child fear indices. Hence we could not perform a *multi-level* meta-analysis. If a study did report multiple child fear outcomes, we chose the statistics in the following order (1) cognitive measure of child fear or avoidance (self-reported fear) and (2) behavioral measure of child avoidance. Only one study assessed child fear with a physiological index (Aktar et al., 2022).

Eleven studies that were included in the meta-analysis assessed parental anxiety. However, only four studies that were included in the meta-analysis reported findings on parental anxiety as a moderator (Aktar et al., 2014, 2018; Bell et al. 2015; Nimphy et al., 2023a). Twelve studies that were included in the meta-analysis assessed child temperament. Five studies that were included in the meta-analysis reported findings on child anxiety disposition as a moderator (Aktar et al., 2014, 2018; Bell et al. 2015; Nimphy et al., 2023a; Remmerswaal et al., 2013).

### Main Results

#### Meta-Analysis

The effect of parental verbal threat expression on child fear reaction was Hedges’ *g* = 1.01 (SE = 0.17, CI [0.67, 1.34], *k* = 17, *p* < 0.0001), indicating that children did display more fear towards the novel stimulus after being exposed to parental threat expressions. There was an indication of heterogeneity (*Q* = 151.82, *p* < 0.0001). The visual inspection of the funnel and forest plots shows some asymmetry and suggest that there might be a small-study effect, since two studies with relatively small samples have the largest effect sizes (Burstein & Ginsburg, 2010; Remmerswaal et al., 2010). However, the two large effect sizes might be explained by the fact that these two studies utilized an experimental design (possibly leading to less noise in the data) and had, according to our quality assessment, higher quality than the mean of the other studies (see Table S1 in supplementary material). Furthermore, the trim-fill method did not indicate missing studies on the left side of the funnel. In sensitivity analysis, we repeated the same analysis with only experimental studies, and only correlational studies. In experimental studies, the effect size of parental verbal threat expression on child fear and avoidance was Hedges’ *g* = 1.26 (SE = 0.25, CI [0.77, 1.75], *k* = 10, *p* < 0.0001), with no indication of missing studies on the left side of the funnel according to the trim-fill method. In correlational studies, the effect size of parental verbal threat expression on child fear and avoidance was Hedges’ *g* = 0.70 (SE = 0.17, CI [0.35, 1.04], *k* = 7, *p* < 0.0001).

**Fig. 2:**
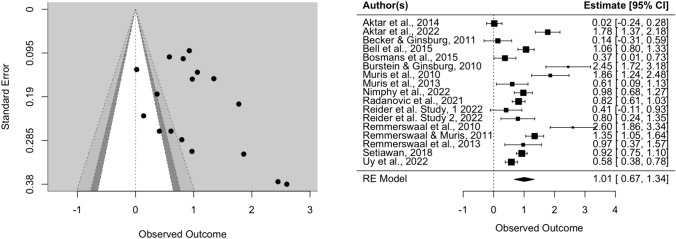
Funnel and forest plots of main effect on child fear

**Fig. 3:**
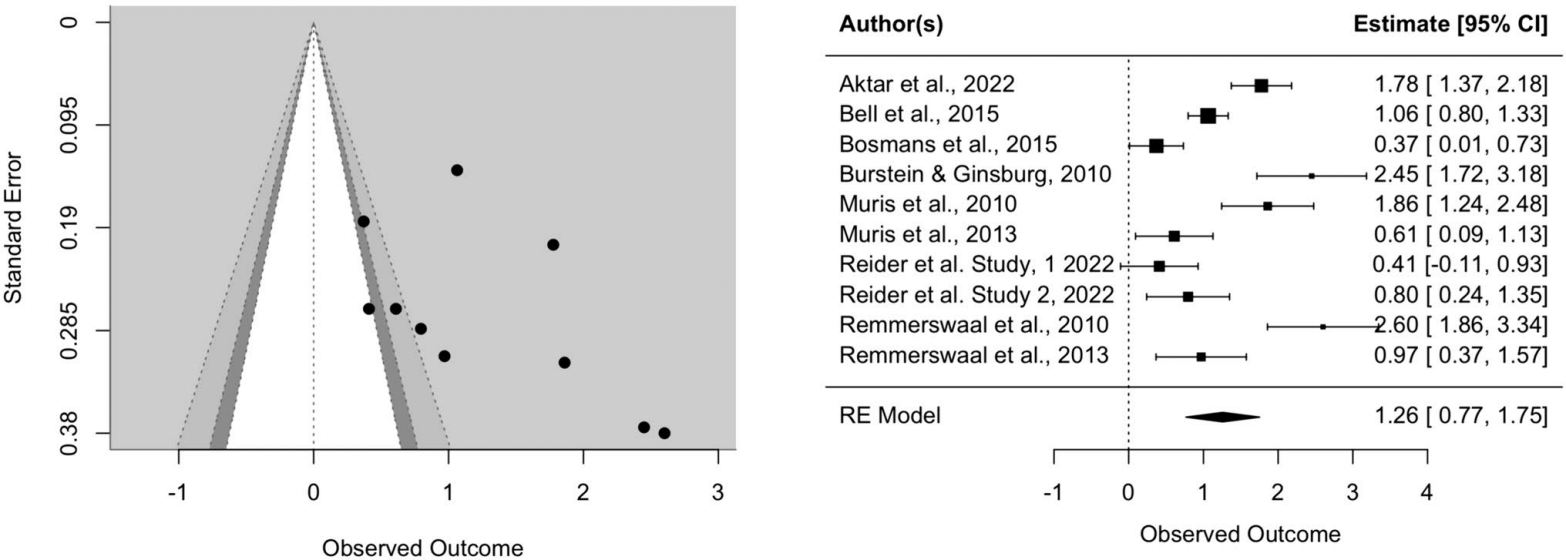
Funnel and forest plots of main effect on child fear in experimental studies only

**Fig. 4:**
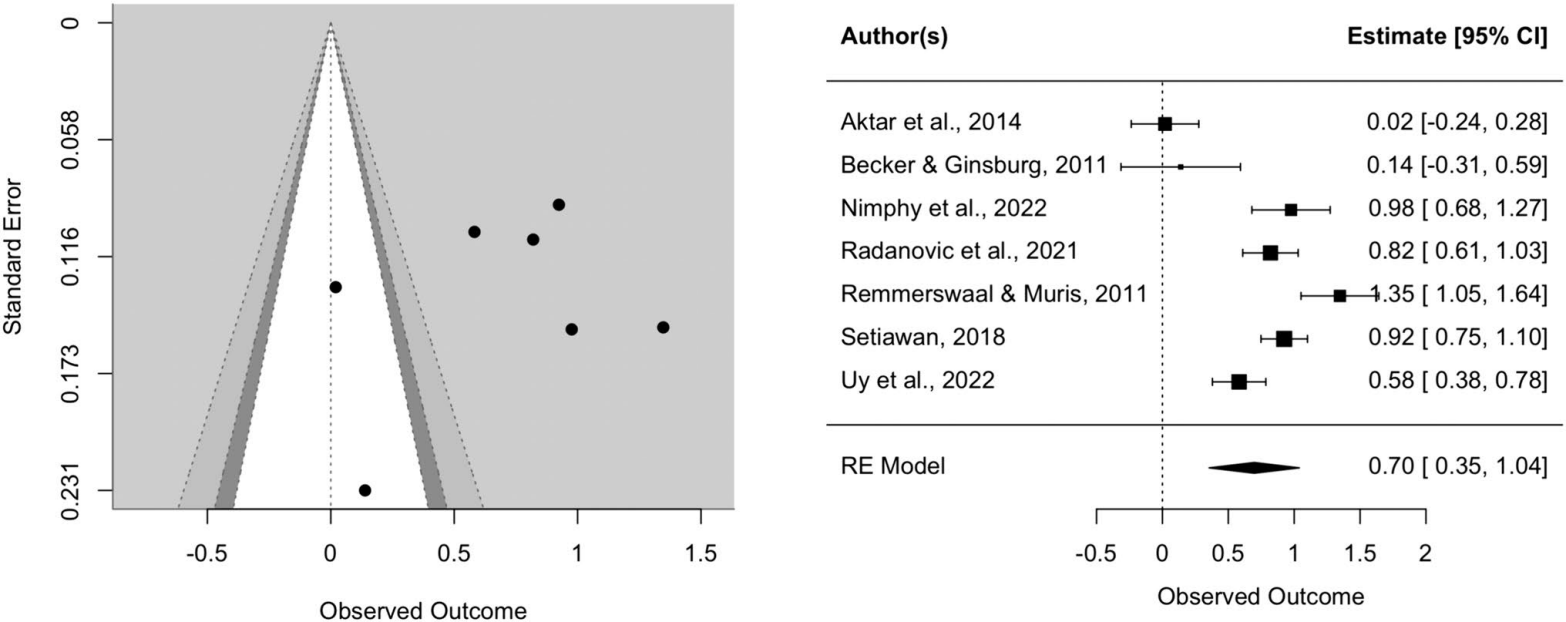
Funnel and forest plots of main effect on child fear in correlational studies only

Funnel and forest plots can be found in Figs. 2, 3, and 4. Inspection of the standardized residuals revealed no outlier (all standardized residuals between 3.29 and −3.29).

#### Systematic Review

A summary of the main findings can be found in Table 3. Based on social fear learning theories (Olsson et al., 2007; Rachman, 1977), we expected that children express more fear and anxiety towards novel stimuli when these stimuli are paired with parents’ fear/anxiety verbal expressions than non-anxious parental verbal expressions. Of the 18 studies reviewed, 13 studies did find an effect on child fear (72%) on at least one of the child fear indices in the expected direction, 4 studies (22%) did not, and 1 study (6%) found an effect in the opposite direction. For 4 studies (22%), findings on different fear indices were mixed. Specifically, they found significant findings on one of the child fear indices (i.e., self-report child fear beliefs) but not on another child fear index (i.e., observed child anxiety).

**Table 3:** Main outcomes and results of moderators on the association between parental verbal communication and fear/avoidance outcomes

Study	Main outcomes	Moderator outcomes	
		Behavioral inhibition/child anxiety	Parental anxiety
Aktar et al. (2014)	Fear/Avoidance: –	Fear/Avoidance: –	Fear/Avoidance^a^: –
Aktar et al. (2018)	Fear: – Avoidance: ↓	Fear: – Avoidance: –	NA
Aktar et al. (2022)	Fear/Avoidance (observed): – Fear (attention bias): – Fear (heart rate): – Fear/Avoidance (self-report): ↑	Fear/Avoidance (observed): ↓ Fear (attention bias): – Fear (heart rate): – Fear/Avoidance (self-report): –	Fear/Avoidance (observed): ↑ Fear (attention bias): – Fear (heart rate): – Fear/Avoidance (self-report): –
Becker and Ginsburg (2011)	Distress: –	N/A	N/A
Bell et al. (2015)	Fear: ↑ Avoidance: ↑	Fear: –	Fear: –
Bosmans et al. (2015)	Fear/Avoidance: – Avoidance: ^b^	N/A	N/A
Burstein and Ginsburg (2010)	Anxiety: ↑ Avoidance: ↑	N/A	N/A
Muris et al. (2010)	Fear/Avoidance: ↑	N/A	N/A
Muris et al. (2013)	Fear: ↑	N/A	N/A
Nimphy et al., (2023a)	Fear: ↑	Fear: –	Fear: –
Radanović et al., (2021)	Fear: ↑	N/A	N/A
Reider et al. (2022) Study 1	Fear/Avoidance: –	N/A	N/A
Reider et al. (2022) Study 2	Fear/Avoidance (snake/spider): ↑ Fear/Avoidance (lizard/turtle): –	N/A	N/A
Remmerswaal et al. (2010)	Fear/Avoidance: ↑	N/A	N/A
Remmerswaal and Muris (2011)	Fear: ↑	N/A	N/A
Remmerswaal et al. (2013)	Fear/Avoidance: – Avoidance: ↑	N/A	N/A
Setiawan et al. (2018)	Fear/Avoidance: ↑	N/A	N/A
Uy et al. (2022)	Fear/Avoidance: ↑	N/A	N/A

↑ = increase in (or presence of) verbal threat communication significantly associated with increase in or higher fear/anxiety (*p* < 0.05)

↓ = increase in (or presence of) verbal threat communication significantly associated with decrease in or lower fear/anxiety (*p* < 0.05)

– = verbal communication not significantly associated with fear/anxiety, if main effect insignificant but 3 or 4-way interaction significant it is labeled as insignificant; NA = interaction not assessed (i.e. only main effect and not interaction with parental verbal fear, or 3-way interactions with another variable), or not assessed at relevant time point/age range

^a^Predictor and outcome not measured in the same paradigm/time point

^b^No information in results section

### Child and Parental Anxiety, and Child Age

#### Meta-Analysis

Child anxiety was not a significant moderator of child fear. The effect of parent responses on child fear did not change as a function of child anxiety (Hedges’ *g* = −0.03, SE = 0.06, CI [−0.15, 0.09], *k* = 4, *p* = 0.64). Parental anxiety was not a significant moderator of child fear. The effect of parent responses on child fear did not change as a function of parental anxiety (Hedges’ *g* = 0.04, SE = 0.06, CI [−0.09, 0.17], *k* = 4, *p* = 0.54). Children’s age was not a significant moderator of child fear. The effect of parent responses on child fear did not change as a function of child age, Hedges’ *g* = 0.05, SE = 0.06, CI [−0.07, 0.17], *k* = 16, *p* = 0.39. Inspection of the standardized residuals revealed no outliers.

#### Systematic Review

A summary of the moderator effects can be found in Table 3. Of the 18 studies reviewed, 5 studies assessed the moderating role of child anxiety (Aktar et al., 2014, 2018, 2022; Bell et al., 2015; Nimphy et al., 2023a; Remmerswaal et al., 2013). Of these 5 studies, none found a significant positive moderating effect of child anxiety in the link between parental verbal threat and child fear. Four studies (80%) did not find a significant moderating effect (Aktar et al., 2014, 2018; Bell et al., 2015; Nimphy et al., 2023a), whereas one study (20%) found an effect in the opposite direction (Aktar et al., 2022).

Of the 18 studies reviewed, 4 studies assessed the moderating role of parental anxiety (Aktar et al., 2014, 2022; Bell et al., 2015; Nimphy et al., 2023a). Of the 4 studies that investigated parental anxiety as a moderator, 1 study (25%) found a significant moderating effect of parental anxiety in the link between parental verbal threat and child fear (Aktar et al., 2022). Three studies (75%) did not find a significant moderating effect (Aktar 2014; Bell et al., 2015; Nimphy et al., 2023a).

Regarding the possible moderating effect of child age, the one study that assessed child age as a moderator found an effect of parental threat on child fear and avoidance, but only in younger children (Uy et al., 2022).

## Discussion

This systematic review and meta-analysis systematically assessed the role of parental verbal threat information in the parent–child transmission of fears. The meta-analytic findings show that parental verbal threat information about novel stimuli can increase child fear—even after a single exposure to these stimuli (Hedges’* g* = 1.26). In line with our systematic review, the meta-analytic findings did not reveal a moderating role of parental and child anxiety levels, or child age in this parent–child transmission of fears to novel stimuli. Below, we discuss each of these findings in turn.

### Child Fear

This systematic review and meta-analysis revealed that parental verbal expressions about novel stimuli are linked to and can increase child fear reactions to these stimuli. These findings align with social fear-learning models (Olsson et al., 2007; Rachman, 1977), and corroborate parental verbal threat information as a causal social fear-learning pathway. The average effect size of the impact of parental verbal threat information on child fear was large (Hedges’ *g* = 1.01 in all studies, Hedges’ *g* = 1.26 in experimental studies only, and Hedges’ *g* = 0.70 in correlational studies only). A recent meta-analysis that systematically assessed the effect (size) of the modeling of parental *nonverbal* fear expressions (also known as vicarious learning) in infancy (Nimphy et al., 2023b) found small to medium effect sizes (Hedges’ *g* = 0.39). Hence, the impact of parental *verbal* fear expressions about novel stimuli appears to be larger on children’s fear of these stimuli, compared to the impact of parental *nonverbal* expressions. While it could be possible that verbal expressions of anxiety are more direct and impactful on children’s reaction than nonverbal expressions, it is important to mention that multiple studies that are included in the current meta-analysis manipulated *both* parental verbal threat information and non-verbal expressions of anxiety. The combined impact of nonverbal and verbal expressions of fear might explain the stronger effect size for our meta-analytic findings on fear learning via parental verbal threat information compared to fear learning via modeling.

Furthermore, in the current meta-analysis, studies predominantly assessed child fear through self-report questionnaires. Exposure to parent verbal threat information might only/to a larger degree impact children’s subjective fear levels, rather than the physiological or behavioral fear components. Since fear indices are often unrelated (Bradley & Lang, 2000), if children report more fear of a novel stimulus, it does not necessarily mean that children would also behave more fearful of the stimulus. Studies that only assess one fear index may not be able to capture the entirety of children’s fear reactions to the novel stimulus. Hence it is important to stress that our conclusions concern self-reported fears, rather than robustly holding across multiple fear indices, i.e., physiological or behavioral indices.

In our meta-analysis, we found a larger effect size on the link between parental verbal threat information and child fear in the experimental studies than in the correlational studies. While experimental studies investigated fear transmission in a lab by manipulating parental verbal information, the correlational studies assessed the relationship between naturally occurring communication of parental threat information and child fear of novel stimuli in daily life. The larger effect size in experimental studies might be explained by the increased control in the lab setting and reduction of the influence of confounding variables. Taken together, findings of our systematic review and meta-analysis revealed that parental verbal expressions about novel stimuli are linked to and can increase child self-reported fear of these stimuli.

### Child and Parental Anxiety Dispositions

Based on susceptibility models, we expected children with higher anxiety levels/dispositions to be more susceptible to environmental stressors such as parental verbal threat information (Belsky & Pluess, 2009; Ingram & Luxton, 2005; Nigg, 2006). Against expectation, our meta-analysis did not reveal a moderating effect of child anxiety levels or disposition on child *fear* (Hedges’ *g* = −0.03). Our systematic review revealed that the majority of the studies (4 out of 5) found no significant effect on child fear, and one in the opposite direction (decrease in avoidance). It could be that child anxiety dispositions, such as temperament make children more susceptible to parental verbal threat information (or nonverbal fearful expressions) in early life, rather than in childhood (see Nimphy et al., 2023b). Moreover, instead of making children more susceptible to parental threat information, child anxiety dispositions in childhood might increase fearful responses to novel stimuli independent of parental information. Lastly, the anticipated moderating effects might not have been detected due to the strength and intensity of the experimental manipulation in most studies. In real life, threat-related information might be less explicit and more ambiguous, compared to the experimental manipulations. For instance, it is possible that children’s anxiety disposition plays a stronger role in fear acquisition if children are exposed to more ambiguous and less explicit verbal information. More ecologically valid designs are needed to investigate the role of anxiety dispositions in parent–child transmission of anxiety (for example, see Muris et al., 2010). Since our interpretation is based on only five studies, more research is needed to investigate whether child anxiety dispositions are a risk factor for heightened fear acquisition after exposure to parental threat information. Nevertheless, until now, our findings suggest that the link between parent verbal threat information and child fear acquisition is not stronger for children with anxiety dispositions.

We explored whether children of parents higher in anxiety disposition are more susceptible to parental verbal threat information. However, our meta-analysis did not reveal a moderating effect of parent anxiety levels on child *fear* (Hedges’ *g* = 0.04). Our systematic review revealed that the majority of the studies (3 out of 4) found no significant effect on child fear. The only study that did find an effect was on a behavioral index of child fear, thus it remains possible that the predominant focus on subjective indices made this less visible/apparent. However, only a limited number of studies investigated the moderating role of parental anxiety in the link between parental verbal threat information on child observed fearful/avoidant *behavior*. Further research is needed to establish whether children of anxious parents might show increased fearful and avoidant behavior after parental verbal threat information compared to children of less anxious parents.

Another explanation for the finding that children of parents higher in anxiety disposition were more susceptible to parental verbal threat information might be that parental anxiety disorders rather than parent’s anxiety levels make children more susceptible to parental verbal threat information. For example, it could be that the repeated exposure to verbal threat information in families with anxious parents creates an anxiogenic environment and contributes to the familial aggregation of anxiety (also named chronic exposure, Perlman et al., 2022). Additionally, anxious parents may be more inclined to endorse or facilitate their children’s anxious or avoidant reactions to novel stimuli and may opt to remove their children from situations where they could get exposed to these stimuli (Fisak & Grills-Tacquechel, 2007). These anxious parents may less frequently use adaptive strategies, such as providing a comforting object, reacting supportively, or demonstrating other problem-solving approaches, for regulating their children’s emotions (Stifter & Augustine, 2019). Consequently, these parental behaviors could potentially over time diminish children’s sense of self-efficacy for self-regulation and elevate their fears (Stifter & Augustine, 2019) and contribute to the heightened fear learning. Another possibility is, that rather than making children more susceptible to parental threat information, parent anxiety dispositions might act more as a risk factor for increased fear and anxiety responses independent of parental verbal information. Given the limited number of studies assessing the moderating role of parental anxiety levels, more research is needed to investigate its role in the parent–child fear transmission in community and clinical samples. Until now, the findings do not support a moderating role of parental anxiety levels in fear acquisition after a single exposure to parental verbal threat information.

### Child Age

We examined if the impact of parental verbal information on children’s fear reactions to novel stimuli may differ across children’s age. We expected that younger children, who may have lower emotion regulation capacity to deal with parental verbal threat information, compared to older children, are more sensitive to this information and show increased fear learning. However, our meta-analysis did not reveal a moderating effect of child age on child fear (Hedges’ *g* = 0.05). Prospective studies, which investigate the parent–child transmission of fears over time, might help illuminate whether fear learning via verbal threat information differs across age.

### Clinical Implications

By investigating social fear learning mechanisms and how they might differ between healthy and at-risk families, we may gain more insight into which specific pathways and factors to focus on in treatment or prevention strategies. In our meta-analysis, we found a large effect of parents’ verbal threat information about novel stimuli on child fear reactions towards these stimuli, independent of child or parental anxiety levels. While fear acquisition via this pathway can be seen as an adaptive response to potentially threatening and novel stimuli, it could be that in *at-risk families*, the exposure to parental verbal threat information in day-to-day life occurs more frequently or intensely, which could strengthen the impact of this fear learning pathway. To prevent child anxiety development via this route, prevention strategies could incorporate psychoeducation on parent-to-offspring social fear transmission. Given the large effects found in the verbal threat information pathway, prevention efforts could potentially target the (repetitive) verbal communication of the parent.

As parental verbal threat information can lead to fear acquisition towards novel stimuli in children, listening to parents’ positive or confident information may reduce or prevent fear acquisition. A recent systematic review assessed if children’s positive modeling (of parents, experimenters, and peers) in experimental studies can reduce or prevent fear acquisition to novel stimuli (Krause & Askew, 2022). Although their conclusions rely mostly on modeling rather than verbal information/instructional learning, from a limited number of studies, it might still be a promising pathway to reduce or prevent children’s fear acquisition to novel stimuli. Ultimately, gaining insight into children’s fear acquisition in developmentally sensitive designs and investigating potential strategies to reduce or prevent parent-to-child fear transmission is crucial to inform treatment and prevention efforts.

### Limitations and Future Directions

This is the first meta-analysis on the effect of parental verbal threat information about novel stimuli on child fear of these stimuli. While this meta-analysis provides a less biased summary of existing studies on the parent–child transmission of fears via verbal threat information, this study still embodies the shortcomings of the individual empirical studies.

First, the studies included in our meta-analysis mainly consist of WEIRD (Western, educated, industrialized, rich, and democratic) samples, specifically predominantly Caucasian families with moderate to high SES (socio-economic status). Considering cultural factors when investigating children’s perception and reaction to parental emotional expressions is crucial (review by Nielsen et al., 2017; Raval & Walker, 2019). To enhance the generalizability of our findings, future research investigating this fear-learning pathway should include more diverse samples, and/or compare this fear-learning pathway across multiple cultural environments.

Second, caution is warranted for the generalizing of our findings to real life parent–child fear transmission. The majority of studies, which are included in this meta-analysis, utilized an experimental design and tested the verbal learning effects in lab-based artificial contexts. While conducting experimental studies on this parent–child fear transmission pathway allows for stronger conclusions on causality (Kazdin, 2021), it may limit the generalizability of the findings to experiences in daily life. Children’s experience with the novel stimuli presented in the lab might not generalize well to their experience outside of the lab. Furthermore, in experimental studies, parents are trained to display specific verbal and nonverbal expressions of anxiety, which might also not represent how parent show fear in daily life. While children can be exposed to one parent’s reaction in the lab, in real life they might get exposed to conflicting emotional reactions from two parents/individuals, successively or simultaneously. These conflicting reactions may alter the child’s response to the novel stimuli. Hence, future research should assess this fear-learning pathway in multiple contexts, as well as investigate naturalistic observations in families with children or parents with an anxiety disorder.

It is also important to note that the majority of studies included in the systematic review and meta-analysis assessed fear reaction to non-social stimuli, such as animals. Thus, more research is needed to assess children’s fear acquisition via parental verbal threat information to social stimuli. Moreover, in multiple studies from our meta-analysis, children were not actually exposed to the novel stimulus. Rather, some studies asked children how they feel about or would react to the stimulus in a hypothetical encounter, or in anticipation of being exposed to the stimulus. Future research could try to disentangle the different effects of parental verbal threat information on children’s fear reaction in anticipation or as a reaction in an actual encounter with the novel stimulus, utilizing multiple fear indices, such as cognitive, behavioral, and physiological indices measured at multiple time points.

## Conclusion

In this meta-analysis, we found a large effect of parental verbal threat information towards a novel stimulus on child fear of the stimulus—even after a single exposure. Parents’ verbal information about novel stimuli matter and can prompt child fear learning. There was no support for the hypotheses that child’s anxiety disposition, child age, or parental anxiety disposition strengthen environmental acquisition of fears via parental verbal threat information.

## Supplementary Information

Below is the link to the electronic supplementary material.

Supplementary file1 (DOCX 21 KB)

## Data Availability

All raw data and materials will be stored online within 3 months after publication to DataverseNL, available upon request from the corresponding author.
